# Dengue fever in a patient with severe haemophilia: a case report

**DOI:** 10.1186/s13104-015-1043-x

**Published:** 2015-03-12

**Authors:** Dilushi Wijayaratne, Priyanga Ranasinghe, Shanaka P Mohotti, Shani Apsara Dilrukshi, Prasad Katulanda

**Affiliations:** University Medical Unit, National Hospital of Sri Lanka, Colombo, Sri Lanka; Department of Pharmacology, Faculty of Medicine, University of Colombo, Colombo, Sri Lanka; Department of Clinical Medicine, Faculty of Medicine, University of Colombo, Colombo, Sri Lanka

**Keywords:** Haemophilia, Dengue, Dengue fever

## Abstract

**Background:**

Dengue fever is the most rapidly spreading mosquito-borne viral disease in the world. Haemophilia A is the commonest inherited bleeding disorder. There is little data on the incidence and outcome of dengue in patients with haemophila. We report a case of a patient with severe haemophila A, presenting with dengue fever, managed at a tertiary care hospital in Sri Lanka.

**Case presentation:**

A 16-year-old Sinhalese male with severe haemophilia A (factor level < 1percent) was admitted to a teaching hospital in Sri Lanka on day 1 of an acute febrile illness, associated with arthralgia, myalgia, vomiting and headache. On admission, he had a tachycardia of 120 beats per minute, and blood pressure of 110/70 millimetres of mercury, with no bleeding manifestations. Baseline investigations revealed leukocyte and platelet counts of 4400 and 241,000 per cubic millimtre, respectively, and a haematocrit of 34.5 percent. Dengue was confirmed later by sero-conversion of the dengue IgM antibody test. Fluid balance, pulse rate and blood pressure were monitored hourly. The haematocrit and platelet counts were checked thrice daily, while he was clinically assessed for bleeding. On day 3 he developed bleeding from a tooth extraction site, with vomiting of dark red blood. His platelet level at that point was 124,000 per cubic millimetre with a haematocrit of 32 percent. Intravenous factor VIII was given to achieve a 100 percent factor correction over twenty-four hours. His platelet count dropped progressively from admission to a nadir of 50,000 per cubic millimetre on day 6. He did not develop clinical evidence of fluid leakage. On day 7 he was discharged after complete recovery.

**Conclusions:**

People with haemophilia may exhibit bleeding from the early febrile stage and at higher platelet levels than most other patients with dengue. Further discussion and research is necessary to decide on the optimal management of these patients, with regard to monitoring and timely treatment with blood products and/or factor correction, in order to prevent dengue-related morbidity and mortality whilst avoiding overtreatment. In endemic areas it is advisable that such patients seek early medical help in the event of an acute fever.

## Background

Dengue fever (DF) is a vector borne viral disease caused by the dengue virus, which belongs to the family filoviridae and genus flavivirus [1]. Currently it is the most rapidly spreading mosquito-borne viral disease in the world [2]. Sri Lanka, is an island nation in the Indian subcontinent that has a population of nearly 20.5 million [3]. DF is endemic in most parts of Sri Lanka, where it accounts for a large proportion of all hospital admissions with acute fever. In 2011 alone 27,162 cases of DF were reported in Sri Lanka, with 246 deaths [4]. Dengue has a wide spectrum of clinical presentations, often with a clinically unpredictable evolution and outcome. Most patients with DF recover following a self-limiting non-severe clinical course, while a small proportion progress to develop severe disease, characterized by plasma leakage with or without haemorrhage [2]. Severe thrombocytopenia and increased vascular permeability are the two major characteristics of dengue hemorrhagic fever (DHF) [5].

Haemophilia A is the most common inherited bleeding disorder, caused by defects in the gene that encodes coagulation factor VIII. It is an X-linked recessive disorder which occurs in approximately 1:5000 males [6]. Patients with pre-existing coagulopathies such as haemophila are probably at a higher risk of bleeding than others. It is likely that such patients may develop bleeding at higher platelet counts than the normal population. There is a scarcity of data on the incidence and outcome of DF in patients with haemophila [7]. We report a case of a patient with severe haemophila A (factor level <1%) presenting with DF, managed at a tertiary care hospital in Sri Lanka.

## Case presentation

A 16-year-old Sinhalese male with severe haemophilia A (factor level < 1%), was admitted to a teaching hospital in Sri Lanka on Day 1 of an acute febrile illness. He had been diagnosed with haemophilia in early childhood, but was not on prophylactic factor VIII therapy. Four days before admission, he had undergone a dental extraction, with 30% factor correction with intravenous factor VIII prior to the procedure. One day before admission he had developed bleeding from the extraction site, which had been treated with 750 IU of factor VIII to achieve a 30% factor correction. On the day of admission he had developed fever with chills and rigors, associated with arthralgia, myalgia, nausea, vomiting and severe frontal headache.

On admission, he was febrile and flushed, with a heart rate of 120 beats per minute and a blood pressure of 110/70 millimetres of mercury. The respiratory rate was 20 per minute, with clear lungs on auscultation. The abdomen was soft, with mild epigastric tenderness. Neurological examination including the fundus examination was unremarkable. There was no evidence of bleeding on clinical evaluation at the time of admission.

Baseline investigations revealed a white blood cell count of 4400 per cubic milimetre (/mm^3^) (Neutrophils 47%; Lymphocytes 41%), while the platelet count and haematocrit were 241,000/mm^3^ and 34.5% (haemoglobin 11.3 g/dl) respectively. The erthyrocyte sedimentation rate, urine full report, serum creatinine, hepatic tranasaminases, prothrombrin time and Chest X-ray were all within normal limits and remained normal during the entire hospital stay. A clinical diagnosis of a possible undifferentiated viral fever or dengue fever (DF) was made based on the history, examination and investigations. Subsequently DF was confirmed by sero-conversion with the dengue IgM antibody test (MAC-ELISA, M Antibody capture- Enzyme Linked Immunosorbent Assay) on the tenth day. Earlier confirmation by antigen detection was not possible due to limited availability of test. He was managed as dengue, according to the national dengue management guidelines, with hourly monitoring of fluid balance, pulse rate and blood pressure [8]. The haematocrit and platelet counts were checked thrice per day. The patient was frequently clinically evaluated to detect the presence of any bleeding.

On day 2 of the illness he complained of severe throbbing headache associated with vomiting. Neurological examination, including fundoscopy, was normal. An urgent non-contrast computerized tomography (CT) scan of the brain excluded the presence of any intracranial bleeding. On day 3 of the fever he developed recurrence of bleeding from the tooth extraction site, together with vomiting of dark red blood. His platelet level at that point was 124,000/mm^3^ with a haematocrit of 32%. Intravenous factor VIII was given to achieve a 100% factor correction over twenty four hours. Omeprazole was administered intravenously for management of possible co-existing gastric erosions or peptic ulcers. Factor correction was discontinued after 24 hours (on day 4) as bleeding had settled and activated partial thromboplastin time (APTT) was in the normal range. Fever resolved by day 5. His platelet count dropped progressively from day 1 to a nadir of 50,000/mm^3^ on day 6. During the course of the illness he did not develop any clinical evidence of fluid leakage or become haemodynamically unstable. On day 7 he was discharged since he was afebrile and clinically well, with a rising platelet count of 69,000/mm^3^.

## Discussion

People with bleeding disorders such as haemophilia, presenting with dengue infection, have an increased risk of bleeding compared to non- haemopliliacs [4]. In a series of 6 cases with DF from Thailand, it was noted that people with haemophilia exhibited bleeding manifestations from the early febrile stage and at higher platelet levels, in contrast to the pattern observed in most other patients with DF [7]. This is possibly due to the lack of factor VIII and/or aggravation of bleeding risk due to the vasculopathy in DF [7] In the case of the current patient, who had a non-severe form of dengue, his recent surgical procedure placed him at higher risk of bleeding. The suspicion of concurrent dengue infection in this patient was crucial to his optimal monitoring and management as serious bleeding manifestations could be expected during the late febrile and critical phases when thrombocytopenia occurs. The current WHO DF guidelines do not emphasise congenital bleeding disorders as a ‘coexisting condition’ that necessitates admission, or provide special guidance on monitoring and managing such patients [2]. Considering the higher risk of bleeding, and the chance that bleeding may occur earlier on in disease than in non-haemophiliacs, closer clinical and haematological monitoring may be required than is recommended for non-haemophiliacs.

Further discussion and research is necessary to decide on the optimal management of these patients, especially with regards to monitoring protocols and the requirement and timing of blood products and/or factor correction. In the current patient, it was decided to achieve a 100% factor correction with the appearance of mild bleeding while monitoring APTT. The place of prophylactic factor correction and its timing in patients with confirmed DF is not clearly understood. While under-correction poses a risk of bleeding, indiscriminate factor replacement places the patient at a risk of side effects, and is extremely expensive [9]. Hence, there is an urgent necessity for evidence-based and/or interdisciplinary consensus-based guidelines on the management of DF in patients with bleeding disorders such as haemophilia. In endemic areas such as in Sri Lanka it is advisable for patients with haemophilia to seek early medical help in the event of an acute fever. As most patients with congenital bleeding disorders are regularly followed up at hospital clinics, education in this regard can be easily achieved.

## Conclusion

People with haemophilia who develop dengue virus infections may exhibit bleeding manifestations from the early febrile stage and at higher platelet levels, compared to the pattern observed in most non-haemophiliac patients with dengue fever. Further discussion and research is necessary to decide on the optimal management of these patients, with regard to monitoring and timely treatment with blood products and/or factor correction, in order to prevent dengue related morbidity and mortality whilst avoiding overtreatment. In dengue endemic areas it is advisable that patients with bleeding disorders such as haemophilia seek early medical help in the event of an acute fever.

## Consent

Written informed consent was obtained from the patient and the patient’s legal guardian(s) for publication of this case report and any accompanying images. A copy of the written consent is available for review by the Editor-in-Chief of this journal.

## Abbreviations

DF: Dengue fever
DHF: Dengue haemorrhagic fever
WHO: World Health Organization
APTT: Activated partial thromboplastin time
IV: Intravenous
IU: International units
PT/INR: Prothrombin time/International normalized ratio
